# Effects of Pro/Prebiotics Alone over Pro/Prebiotics Combined with Conventional Antibiotic Therapy to Treat Bacterial Vaginosis: A Systematic Review

**DOI:** 10.1155/2022/4774783

**Published:** 2022-04-21

**Authors:** Roghayeh Afifirad, Amir Darb Emamie, Rezvan Golmoradi Zadeh, Parisa Asadollahi, Roya Ghanavati, Atieh Darbandi

**Affiliations:** ^1^Department of Microbiology, School of Medicine, Tehran University of Medical Sciences, Tehran, Iran; ^2^Department of Pathobiology, School of Public Health, Tehran University of Medical Sciences, Tehran, Iran; ^3^Department of Microbiology, School of Medicine, Iran University of Medical Sciences, Tehran, Iran; ^4^Microbial Biotechnology Research Centre, Iran University of Medical Sciences, Tehran, Iran; ^5^Department of Microbiology, Faculty of Medicine, Ilam University of Medical Sciences, Ilam, Iran; ^6^Behbahan Faculty of Medical Sciences, Behbahan, Iran

## Abstract

**Background:**

Bacterial vaginosis (BV), caused by an imbalance in the vaginal microbiota, can be treated and prevented by probiotics. Pregnant women with BV can experience premature labor and spontaneous abortions. Probiotics and prebiotics promote the proliferation of beneficial microorganisms, alter the composition of the vaginal microbiota, and prevent intravaginal infections in postmenopausal women. In addition to reducing infection symptoms, pre/probiotics can also help prevent vaginal infections.

**Materials and Methods:**

A systematic review was conducted on studies from 2010 to 2020 to determine the efficacy of pre/probiotics on the treatment of BV in pregnant and nonpregnant women. The databases Medline, Scopus, Embase, and Google Scholar were systematically searched using the following keywords: “bacterial vaginosis,” “probiotics,” “prebiotics,” and “synbiotics.”

**Results:**

A total of 1,871 articles were found in the initial search, and 24 clinical trials were considered eligible. In studies comparing the effects of pre/probiotics and placebos with or without antibiotic therapy in patients with BV, significant differences in clinical outcomes were observed. Probiotics reduced the levels of IL-1*β* and IL-6, as well as the overall Nugent score and Amsel's criteria for restitution of a balanced vaginal microbiota. In addition, probiotics can reduce the vaginal colonization of Group B streptococci among pregnant women. In subjects treated with probiotics, BV cure rates were higher than those in subjects treated with antibiotics. There were no additional adverse events.

**Conclusion:**

Pre/probiotic regimens, when used for BV treatment, are usually safe and can exhibit long-term and short-term benefits. In order to prove the benefits of pre/probiotics in BV treatment, additional high-quality research is required.

## 1. Introduction

Bacterial vaginosis (BV) is one of the most common diseases among women of childbearing age [1]. The prevalence of BV varies from 5% in Asia and Australia to 59% in Southern and Eastern Africa [2, 3]. In pregnant women, one of the particular groups of interest with elevated BV adverse events (AEs) rates, the BV prevalence ranges from 8% to 51% [4]. The prevalence of BV among Iranian women is estimated at 18.9% [5]. While BV is considered a mild disease, it can be associated with uterine infections and adverse pregnancy outcomes [6, 7]. In addition, there are BV-associated complications such as pelvic inflammatory disease (PID), which causes preterm premature rupture of the membranes (PPROM), miscarriage, and premature delivery [8–10]. Asymptomatic or symptomatic BV is associated with several sexually transmitted infections (STIs), including *Chlamydia trachomatis*, *Neisseria gonorrhoeae*, HSV-2, and an increased risk of increased risk HIV-1 acquisition [1]. Researchers have recently confirmed the link between BV and human papillomavirus (HPV) [11]. Treatment of BV with first-line antibiotics such as metronidazole and clindamycin causes 80% improvement in symptoms of patients after 4 weeks of treatment [12]; however, recurrence has been observed in 40% to 50% of cases after 12 months after antibiotic treatment [13]. Although these antibiotics are safe, several side effects have been reported with their use (Oduyebo et al., 2009). Clindamycin and metronidazole have been known to cause gastrointestinal problems, including nausea, vomiting, and abdominal pain (Menard, 2011). Another problem with common BV treatment strategies is that they do not consider the impact of vaginal microbiota disruption on the disease occurrence, an event deteriorated even more by antibiotics [14, 15]. BV treatment should focus on eliminating patients' clinical symptoms, reducing the recurrence rate, and regenerating the vaginal microbiome. BV may be associated with a specific composition and metabolic pathway of the vaginal microbiome, and probiotics may reduce the recurrence rate of the condition [16]. When administered in appropriate amounts, probiotics provide many therapeutic benefits [17]. Probiotics have been used in addition to conventional treatment methods to treat BV. The primary mechanism of treatment for BV with probiotics is reestablishing the vaginal microbiome [18]. Various studies have shown that the reduction of vaginal hydrogen peroxide-producing *Lactobacillus* spp. such as *L*. *crispatus*, *L*. *iners*, and *L*. *gasseri* is closely associated with BV occurrence. Moreover, *Gardnerella vaginalis* overgrowth disrupts the vaginal microbiome by increasing the vaginal pH [19]. *G*. *vaginalis* is closely associated with the colonization of anaerobic organisms and the development of BV symptoms [20]. The effect of metronidazole/clindamycin combined with pre/probiotics or probiotics alone on treating BV has been studied recently. However, it has not been adequately demonstrated that pre/probiotics effectively treat BV. In this review, pre/probiotics were assessed with and without antibiotics, for example, metronidazole and clindamycin, for treating BV.

## 2. Methods

### 2.1. Guidelines

Preferred Reporting Items for Systematic Reviews and Meta-Analysis (PRISMA) guidelines were used for this review [21]. This study has been registered in PROSPERO (the international prospective register of systematic reviews), CRD42021243764 (https://www.crd.york.ac.uk/PROSPERO/display_record.php?Recordid=243764). Our literature search was commenced before registration, and data extraction was underway (but not completed) when registered.

### 2.2. Search Strategy

Four international information databases (Medline, Scopus, Embase, and Google Scholar) were searched to retrieve studies published from 2010 until 2020 using the keywords “bacterial vaginosis” and “probiotics.” The keywords were also used in combination using the Boolean operator (or equivalent operator for the database) to improve the results. The search strategy was adapted to the particularities of each database.

### 2.3. Eligibility Criteria

Randomized controlled trials (RCTs), published in English that compared human supplementation with probiotics products and reported objective measurements of bacterial vaginosis, were included in this review. Studies without detailed information regarding the probiotics interventions and those without originality (reviews, short communications, case studies, abstracts without full texts, and book chapters) were excluded from consideration.

### 2.4. Data Collection and Analysis

Articles were searched and screened by two independent authors, analyzing the titles and abstracts as the initial step of the selection process. Finally, the authors read the studies to see if they met the eligibility requirements. The selected studies were independently coded and extracted by two reviewers. A third independent author reviewed the process and clarified any discrepancies. Reference lists of the included publications were also investigated to identify any articles missed. Publications cited in more than one database were only included once. To collect and organize references, EndNote was used as an auxiliary tool. The following relevant data were extracted from each study into a Microsoft Excel file: first year of publication, location of research, sample characteristics (type and size), experimental conditions (study design and protocol), and probiotic intervention (strain type, dose, and duration).

### 2.5. Risk of Bias

Two independent reviewers used Joanna Briggs Institute (JBI, 2014) Critical Appraisal Checklist tool [22] to evaluate the quality of the eligible RCTs. Each study received a score ranging from 0 to 13 points. Finally, the studies with high-quality papers were included in the current review (Supporting information Table 1).

## 3. Results

**Figure 1:**
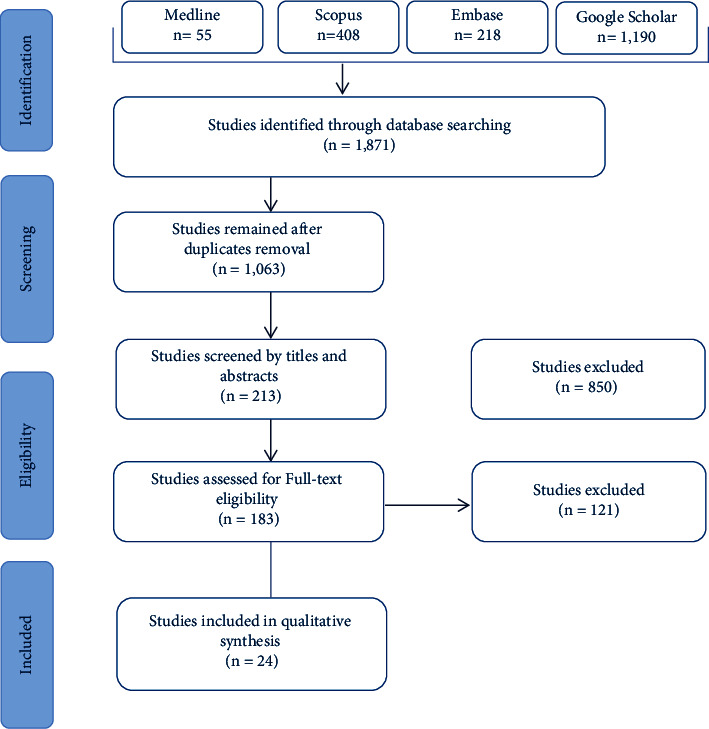
PRISMA flowchart of articles for the systematic review.

The search strategy and article exploring process are shown in Figure 1. A total of 1,871 articles were excluded based on their titles and abstracts, following which 183 articles were retained for detailed full-text evaluation. After the full-text analysis, 24 studies were found to examine the efficacy of probiotics and/or prebiotics in combination with conventional antibiotic therapy in treating or preventing BV and were, therefore, considered for further analysis.

**Figure 2:**
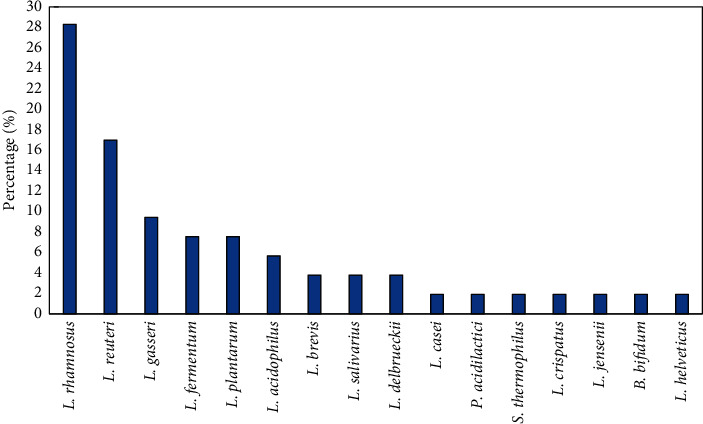
Frequency of the probiotic species used for the treatment of women with BV in 24 trials.

Table 1 summarizes the characteristics and subgroups of the participants in the articles included in this paper. The results of different clinical trials evaluating the efficacy of pre/probiotics with or without antibiotics on BV treatment are presented in Tables 2 and 3. Most of the studies, which examined the effects of probiotics on BV treatment, were carried out in Germany and Indonesia (3 out of the 24 studies and 396 out of the 8,242 participants), followed by Australia and Poland, each with two studies. The specimens, including vaginal/cervicovaginal swabs, cervicovaginal lavage (CVL), blood, serum, and histopathology samples, were obtained from subjects with a mean age of 32.1 ± 6.4 (ranging from 18 to 50 years). Among the 24 studies, 10 examined the effect of probiotics in combination with antibiotics, 12 assessed the effect of probiotics, and 2 assessed the effect of prebiotics without antibiotics on the BV treatment. A total of 16 different probiotic species were administered once, twice, or three times daily at doses of 1 × 10^4^ to 6 × 10^9^ colony forming units (CFUs). The average dose of probiotics was 1.35 × 10^9^ CFU.  Figure 2 shows the frequency of probiotics administered for BV treatment in different studies. Based on the results of different studies, *Lactobacillus rhamnosus* was the most common probiotic used (28.3%), followed by *Lactobacillus reuteri* (16.9%) and *Lactobacillus gasseri* (9.4%).

Clinical trials assessing the effects of pre/probiotics on the treatment of bacterial vaginosis: Table 2 shows the results of 14 RCTs examining the effects of pre/probiotics alone on BV-affected women, among which one study used only one probiotic strain to treat genitourinary infections, and 13 studies used two or more probiotic strains.

In the study carried out by Hemalatha et al. [25], CVL was collected to measure the concentrations of IL-1*β*, TNF-*α*, and IL-6 by ELISA. In both treatment arms, the activity of NSMASE (neutral sphingomyelinase) was also quantified. There were no side effects reported for the probiotic vaginal tablet. It was demonstrated that the pre/probiotic treatment effectively restored the normal vaginal microbiota in 32 % of the women, and 47% of the women had an improved Nugent score (*p*=0.0001). However, 20% of the subjects could not clear up BV 8 days after treatment (*p* > 0.05). A significant reduction in the IL-1*β* (*p* < 0.001) and IL-6 (*p*=0.015) levels was observed after treatment with lactobacilli, but no significant change was observed in the TNF-*α* levels. Indarti Budidarmo [26] found that the eradication rate of BV was 56% in the treatment group and 56% in the control group (*p*=0.77). Also, the satisfaction level (score ≥67) was higher in the placebo group compared to the probiotic group, while the difference between the two groups was not statistically significant (*p*=0.65).

Vujic and colleagues [33] tested in their study the effectiveness of probiotics versus placebo in otherwise healthy women with BV. In the placebo group, 26.9% of the subjects regained a balanced vaginal microbiota, while 61.5% did so in the probiotic group (*p* < 0.001). In the probiotic group, more than half of the urogenital microbiota was still present after 6 weeks, but only about half of the microbiota was maintained in the placebo group (*p* < 0.001).

In a 15-day study, Russo et al. [30] tested in their study the effectiveness of probiotics versus placebo in otherwise healthy women with BV. In the placebo group, 26.9% of the subjects regained a balanced vaginal microbiota, while 61.5% did so in the probiotic group (*p* < 0.001). In the probiotic group, more than half of the urogenital microbiota was still present after 6 weeks, but only about half of the microbiota was maintained in the placebo group (*p* < 0.001).

In a study by Tomusiak et al. [31], the administration of probiotics contributed to a significant decrease in both vaginal pH (*p* < 0.05) and Nugent score (*p* < 0.05) and a significant increase in the abundance of *Lactobacillus* spp. (*p* < 0.05). A total of 82% of women taking the drug at visit III and 47.5% of those at visit IV had *Lactobacillus* strains originating from probiotic capsules. Acute AEs were not reported.

As mentioned above, the use of probiotics is effective in BV treatment in women who are not pregnant. Six trials investigated the effects of probiotics on pregnant women with BV who had Group B *Streptococcus* (GBS) colonization and disrupted urogenital microbiota, based on Table 2. Additionally, the effects of probiotic administration in asymptomatic pregnant women have been examined. Gille et al. [23] assessed the effects of probiotics or placebo on BV treatment among pregnant women with 12 weeks of gestation. There was no significant difference in the proportion of normal vaginal microbiota between the two groups after the treatment (*p*=297).

In a trial carried out by Krauss-Silva et al. [27], 4,204 pregnant women with no history of premature births were screened. The probiotics tested failed to prevent preterm birth.

Ming Ho et al. [28] conducted a study in which 110 pregnant women with vaginal and rectal colonization of GBS were treated orally with two placebo capsules or two probiotic capsules for four nights. On admission for delivery, participants' feces and rectal samples were examined once again for GBS colonization. Twenty-one women in the probiotic group (42.9%) and nine women in the placebo group (18.0%) experienced a change in colonization between these periods (*p*=0.007). Women taking oral probiotics were found to have lower rates of GBS colonization.

In another trial by Olsen et al. [29], 34 GBS-positive women at 36 weeks of pregnancy were evaluated. Vaginal GBS rates did not differ significantly between the control and intervention groups (*p* > 0.05). Evaluation of 16 women who had completed 14 days or more of probiotics administration (*n* = 6) indicated no difference in the vaginal GBS load between the probiotic and control groups. Probiotics significantly increased the number of vaginal commensals (*p*=0.048).

Eighty-six pregnant women with an intermediate BV Nugent score at 13 weeks of pregnancy were treated daily with lactobacilli and placebo. At 28 weeks of pregnancy, Nugent scores returned to normal in 30% of the women in both groups (*p* > 0.05) and remained unchanged until 35 weeks (*p* > 0.05). Most subjects expected a positive pregnancy outcome. At 13 weeks of pregnancy, 93 bacterial species were detected by PCR and DNA sequencing, the most abundant of which were *L*. *iners*, *L*. *crispatus*, *G*. *vaginalis*, and *Atopobium vaginae*. Shannon Diversity Index was not different between the probiotic and placebo groups at 13, 28, or 35 weeks of pregnancy. The probiotic group had higher IL-6 levels at 28 weeks, whereas the placebo group had lower IL-10 levels at 35 weeks (*p* > 0.05).

In the study by Hussain et al. [24], participants were given probiotics or placebo once a day throughout pregnancy. In the placebo and probiotic groups, BV rates were the same at 18–20 weeks of gestation (15%). Among women colonized with the probiotic strains, the proportion of *E*. *coli*, GBS, or other vaginal microbiota was not different. Vaginal bacterial diversity or composition was not different between the probiotic and placebo groups during 9–14 or 18–20 weeks gestation.

Participants in the Barthow et al. [36] trial, expecting infants with a high risk of allergy, were randomly assigned to receive *L*. *rhamnosus* HN001 or placebo until delivery and six months after delivery. The presence of BV and GBS was investigated. This study showed that supplementation with *L*. *rhamnosus* HN001 during pregnancy could reduce BV rate (*p* < 0.05) and vaginal carriage of GBS (*p* < 0.05) before childbirth.

In 2 clinical trials, the effects of probiotics were evaluated following conventional antibiotic treatment in women with BV. In one of these trials, 36 women aged ≥18 years with a stable menstrual cycle or menopause were diagnosed with BV based on the Amsel criteria. Oral metronidazole was administered, after which four weeks were spent consuming either probiotic (125 g of probiotic yogurt) or a placebo. After 4 weeks of intervention, there were no BVs in the probiotic group, while 6 of the 17 participants in the control group still had BV (*p*=0.018). After the intervention, Amsel's score in the probiotic group decreased by 4.0 points versus 2.0 points in the control group (*P*=0.038). In addition, both vaginal discharge and odor (Amsel criteria 2 + 3) significantly decreased after 4 weeks in both the probiotic and the control groups (*p*=0.05 and *p*=0.001, resp.). As compared to the control group, the Nugent scores were decreased by 5.5 (*p*=0.0158) in the probiotic group. In a similar study by Ehsrom et al. [35], RAPD analysis was also used to assess probiotic strains. Eighty-nine percent of women receiving probiotics showed the vaginal presence of these strains following 2-3 days of administration, while 0% in the placebo group showed probiotic vaginal colonization (*p* < 0.0001). More than 50% of women in the probiotic group were colonized by at least one LN strain after one menstruation. After six months, 93% of the colonies were still present. Three-quarters of women who received probiotics were cured of BV following 2-3 days of administration (placebo: 83%) and 78% by the first menstruation (placebo: 71%). Following administration (*p*=0.03) and during the second menstruation (*p*=0.04), the intervention groups experienced less malodorous discharge.

Clinical trials assessing the effects of pre/probiotics combined with antibiotics on the treatment of bacterial vaginosis: RCTs assessing the effects of pre/probiotics combined with antibiotics on the treatment of BV are shown in Table 3. The effects of single-strain probiotics after conventional antibiotic treatments were evaluated in four RCTs. Three RCTs utilized well-characterized and well-selected lactobacilli. One study reported the drugs administered, but the other three did not.

One trial compared the efficacy of lactobacilli combined with 0.03 mg oestriol against metronidazole for BV treatment. In the short term, lactobacilli plus oestriol had similar effects as metronidazole in treating BV, but after a month, the efficacy of lactobacilli plus oestriol was reduced. Heczko et al. compared the effects of metronidazole plus probiotics against a placebo. BV was confirmed by bacterial culture and Nugent score in 241 participants. Probiotics extended the time of BV symptoms relapse by up to 51% (*p* < 0.05) compared to placebo and metronidazole. Probiotics also reduced and maintained the low vaginal pH and Nugent score and increased the vaginal *Lactobacillus* count following the standard antibiotic treatment. In a different trial, 150 women were studied to assess the diversity and richness, as well as the efficacy of the vaginal microbiome. The study included 30 healthy participants, 30 controls with BV, and 30 patients treated with antibiotics or probiotics. On days 5 and 30, probiotic-treated subjects (*p*=0.62) had a higher cure rate than those treated with metronidazole (*p*=0.01). In another study, Palma et al. [45] found a higher chance of solving HPV-related cytological anomalies in long-term probiotic users (79.4%) compared to those in short-term probiotic users (37.5%) (*p*=0.041). Compared to long-term lactobacilli users, 11.6% of the short-term probiotic users had their HPV cleared up, as evidenced by a negative HPV DNA test (*p*=0.044).

In Wijgert et al.'s [44] study, 17 HIV-negative, nonpregnant BV females were entered into the trial. BV (Nugent 7–10) incidence was 10.18 per person-year in the control group and lower in the metronidazole (1.41/person-year; *p*=0.004), Ecologic Femi+ (3.58/person-year; *p*=0.043), and Gynophilus LP groups (5.36/person-year; *p*=0.220). Hakimi et al. [41] performed a comparison of the effects of oral metronidazole tablet and prebiotic intravaginal gel on the treatment and recurrence of BV. Neither the personal or social characteristics nor the clinical and laboratory markers differed significantly between the two groups. BV treatment was improved by the adjuvant administration of the prebiotic vaginal gel. In another study [46], the effects of glucomannan hydrolysates (GMH) and BV Gel were examined on pregnant BV patients. Flow cytometry was used to detect Treg cells in peripheral blood mononuclear cells (PBMCs) of ten thousand total CD4 + CD25 + Foxp3 cells. Also, TGF-*β* levels were observed using ELISA. The results of the analysis showed that the GMH and BV gel were able to reduce Nugent scores (*p* <  0.001) and increase Treg cell presentation (*p*=0.001) and TGF-*β* levels (*p* < 0.05) among BV patients. In contrast to their previous findings, Bradshaw et al. [13] found that combining the first-line antibiotic therapies with a long-term vaginal probiotic did not reduce BV recurrence in women. Studies have been done to determine whether or not BV can be prevented by colonizing the vaginal area with lactobacilli. Study participants with BV had 8.3% more probiotics isolated from their stool compared to the control antibiotic group (*p*=0.041). There was no difference between the BV group and those given triple oral antibiotic at the 1st or 6th month of treatment (*p* > 0.05). The 1st or 6th-month BV cure rates did not correlate with the frequency of the isolated *Lactobacillus* strains in the probiotic group. The effects of adding probiotic supplements to the standard antibiotic therapy on BV recovery were evaluated by Hamid et al. [40]; the BV recovery rate was not significantly different between the probiotic group and the metronidazole (with or without oral probiotics) group. The two groups did not show any significant differences in the vaginal pH, KOH test, or clue cell counts after the therapy (*p* > 0.05).

## 4. Discussion

In this systematic review, 24 RCTs with 8,242 women participants were evaluated to come to a conclusion about the efficacy of probiotic monotherapy and combination therapy on bacterial vaginosis. It remains unclear whether the initial pathogenic event is the overgrowth of anaerobes or the primary diminution of lactobacilli. Several trials (14 RCTs) have shown that BV patients who receive probiotics alone have significantly higher cure rates than those receiving placebo and also improve the microbial pattern in vaginal dysbiosis through the following mechanisms: (1) maintaining a normal vaginal pH by producing lactic acid, (2) inhibiting the growth of pathogens by degrading the epithelium glycogen, and (3) competing with pathogens for adhesion sites by producing bacteriocins, biosurfactants, and H_2_O_2_. Some articles have confirmed that certain strains of vaginal lactobacilli are able to produce H_2_O_2_ which inhibits the adherence of *G*. *vaginalis* to the epithelial cells [47, 48] and (4) have an immunomodulatory effect. Probiotics stimulate the immune defense system to produce the cytokines IL-10 and a decrease in serum level of TNF-*α* [49]. On the other hand, Hemalatha et al. [25] found a significant reduction in the IL-1*β* and IL-6 vaginal levels following treatment with lactobacilli, suggesting that lactobacilli can have anti-inflammatory effects and can cure BV. Moreover, lipoteichoic acids on the cell wall of *Lactobacillus* spp. can block the inflammatory response induced by the lipopolysaccharide by competitively binding to CD14 [19, 50].

There were substantial variations among the pre/probiotics interventions, such as variation in dosage, administration routes, therapy duration, prebiotics type, or probiotics strain and species, which make comparisons across different trials difficult. Even commercially available products might have variations in their ingredients. The use of unique commercially available probiotic products as standardized interventions may solve this issue, but the accessibility of these products may be limited to certain regions and periods.

None of the RCTs have reported significant adverse effects. Since probiotics are not systemically absorbed, they are generally regarded as safe for healthy people [17, 51]. A multicenter RCT [33] has reported that oral or intravaginal probiotics can be used as side-effect-free alternative treatments for BV. Some publications have suggested that the administration of probiotics by any routes at doses between 10^9^ to 10^11^ CFUs can be efficient for BV treatment [52]. Regarding the results of different trials, the most commonly used probiotic species was *L*. *rhamnosus,* and the administration of a cocktail of different probiotic species was more effective than probiotic monotherapies. Both the intravaginal and oral administration of pre/probiotics were associated with significant improvements in the cure rate of BV. Even though the intravaginal route seems to be the preferred route by the many RCTs, the ideal route of delivery for pre/probiotics still remains controversial. Some studies indicated that orally applied lactobacilli may positively influence vaginal health [53]. Regarding the oral route of administration, it is critical to consider the viability of the probiotics strains under high concentrations of gastric acid and bile salts as well as the time lactobacilli can reach and colonize the vagina, which appear to be different person to person [53]. Marcotte et al. [43] illustrated that the efficacy of probiotics was consistent with the short-term efficacy of antibiotics and that the long-term efficacy of probiotics significantly prevented BV recurrence [54, 55].

The results of several trials have shown that pre/probiotics combined with metronidazole (12 trials; 994 populations) or clindamycin (3 trials; 350 populations) are more efficient in reducing BV recurrence rate compared to metronidazole or clindamycin alone. Antibiotic therapy on BV has always been argued to cause high recurrence rates, which might be due to several factors, including the fact that some BV is causing microbes to possess genes that repair the antibiotic-induced DNA damage and hence lead to BV recurrence. Pre/probiotic adjuvants can help prevent this event since pre/probiotic + antibiotic combination can aim at different targets in BV-associated microbes and multispecies BV biofilm, making it difficult for the microbes to counteract the effects. Authors of a Cochrane Review have found that probiotic interventions provide beneficial effects on BV treatment when given in combination with metronidazole [56]. Moreover, a clinical cohort study illustrated that the intravaginal administration of *L*. *delbrueckii* subsp. *lactis* DM8909 is as effective as metronidazole in the treatment of BV, which might be associated with reestablishing the normal vaginal microbiota by this probiotic strain. The present systematic review demonstrates that pre/probiotics might increase the cure rate of BV and could be administered as a cotreatment along with or as a substitute for the conventional antibiotic treatment. There are serious concerns regarding the effects of metronidazole and clindamycin on the growth inhibition of *Lactobacillus* spp. and hence the vaginal dysbiosis following BV antibiotic treatment. High concentrations of metronidazole, that is, between 1000 and 4000 mg/ml, can partially inhibit the growth of *Lactobacillus* spp., while concentrations of 5000 mg/ml can completely suppress the growth of these species [57–59]. Some studies have reported that concentrations between 128 and 256 mg/ml can stimulate the growth of *Lactobacillus* spp. [59]. Carlstedt-Duke et al. [60] have observed a minimal influence of clindamycin on the growth of lactobacilli when employed simultaneously to restore the gut normal microbiota of rats. Of note, antibiotic resistance in the lactobacilli strains was found not to be associated with the extra chromosomal elements since plasmids were not found in these strains [58]. This observation would indicate a low chance of antibiotic resistance transmission to pathogenic microorganisms.

The utilization, route of administration, and timing of antibiotics in relation to the probiotic's intervention varied significantly among investigations. By applying probiotics and antibiotics simultaneously, antibiotics might induce inhibitory effects on the probiotics within the gastrointestinal tract. It is therefore recommended to give antibiotics and probiotics separately within intervals of 2 to 4 hours [61, 62].

The vaginal microbiota in pregnant women undergoes subtle changes and may influence several aspects of pregnancy outcomes. Furthermore, the effect of probiotics on BV-associated microbiota dysbiosis during pregnancy remains unknown. In this review, 7 RCTs provide evidence of pre/probiotics supplementation as prevention and improvement of the BV treatment in pregnant women. Yefet et al., Krauss-Silva et al., and 4 RCTs in Table 2 found that oral probiotic administration colonized the vagina, although the rate of colonization was lower than that in nonpregnant ladies [27, 29, 36, 53, 63, 64]. There are some conceivable explanations for that. This might be due to a slower movement of the digestive tract among pregnant women (due to hormonal changes), which subsequently slows down the movement of Lactobacilli through the intestinal tract. In such a case, it may take longer to show vaginal colonization. Also, oral administration of probiotics may have a positive influence on the vaginal microbial ecosystem through competitive inhibition of pathogens or production of postbiotics [63].

### 4.1. Strengths and Limitations

Compared with the former systematic reviews [17, 19, 53, 65], there are some advantages to the present review; a large number of studies have been reviewed since 2010, with a low risk of bias (24 RCTs with 8,242 patients) which makes the outcomes more valid and reliable. The present study also investigated a large number of factors and outcomes, including the type of intervention (probiotics or synbiotics), dosages, the length of intervention, and follow-up period for prevention or treatment of bacterial vaginosis. Nonetheless, there are some limitations to this study. The small number of available trials, small sample size, variation in the methods and probiotics preparation protocols, types of species, number of probiotic strains, the dosage of probiotics used and mean age of participants among trials, different length of the interventions used, and the follow-up duration as well as exclusion of unpublished study data can be a confounding factor for pooling the studies.

## 5. Conclusions

Given the imperative to reduce antibiotic resistance by decreasing antibiotic therapy, alternative approaches are needed to treat and prevent BV. In several trials that used pre/probiotics in combination with antibiotics, probiotics were rendered effective as an adjunct treatment. In conclusion, probiotics may have a positive effect on the treatment of female bacterial vaginosis. However, further research is needed to validate the beneficial effects and safety of probiotics in the prevention or treatment of bacterial vaginosis.

## Figures and Tables

**Table 1:** Summary of subgroups' characteristics in studies assessing the effects of pre/probiotics for the treatment of bacterial vaginosis

Subgroups	Patients (*N*)	Trials (*N*)
BV diagnostic standards		
Amsel's criteria	3,842	14
Nugent's criteria	4,400	10
Route of intervention		
Oral	3,711	12
Vaginal	4,531	12
Regimens combined with pre/probiotics		
Clindamycin	450	1
Metronidazole	1,256	10
Pregnancy status		
Pregnant	5,182	7
Nonpregnant	3,060	17
Follow-up duration		
Short term (≤ 1 month)	2,940	11
Long term (>1 month)	5,484	13

Total number of patients in all the 24 included trials: 8,242.

**Table 2:** Clinical trials assessing the pre/probiotics effects on the treatment of bacterial vaginosis.

Reference	Region	Study design	Participant's characteristics	No. of participants, mean age (SD)	Probiotics	Administration route	Dose (CFU)	Intervention	Control used/therapy duration	Outcomes
Gille et al. (2016) [23]	Germany	RDTPCT	BV/pregnant	320, 33	*L*. *rhamnosus* GR-1; *L*. *reuteri* RC-14	O	1 × 10^9^	1 capsule/o.d./8 wks	Lactose/o.d./8 wks	Oral probiotics may be suitable for implementation in antenatal care but, as administered here, did not affect vaginal health during midgestation
Husain et al. (2019) [24]	UK	RDBPCT	BV/pregnant	304, 31.2 (5.3)	*L*. *rhamnosus* GR-1; *L*. *reuteri* RC-14	O	2.5 × 10^9^	1 capsule/o.d./42 wks	Excipients capsules/42 wks	Oral probiotics taken from early pregnancy did not modify the vaginal microbiota
Hemalatha et al. (2012) [25]	India	RDB	BV/sexually active	67	*L*. *brevis* CD2; *L*. *salivarius* subsp. *salicinius*; *L*. *plantarum*	V	10^9^	1 tablet/o.d./8 d	1 pH lowering vaginal tablet/o.d./8d	Probiotics prevented BV better than pH tablets in healthy subjects. Lactobacilli reduced IL-1*β* and IL-6 vaginal cytokines. Lactobacilli-containing tablets can cure BV and reduce vaginal inflammation
Indarti et al. (2918) [26]	Indonesia	RDBPCT	BV, VVC, trichomoniasis, or combined	50, 35.1 (6.6)	*L*. *rhamnosus* GR-1; *L*. *reuteri* RC-14	O	2.5 × 10^9^	1 probiotic/o.d./4 wks	Identical placebo/4 wks	There was no clinical and statistical difference in the proportion of cure rate and the level of satisfaction in patients of probiotics and placebo groups after treatment
Krauss-Silva et al. (2011) [27]	Brazil	RCT	Asymptomatic pregnant women	644, NR	*L*. *rhamnosus* GR-1; *L*. *reuteri* RC-14	O	>10^6^	1 capsule/b.i.d./6–12 wks	Identical placebo/6–12 wks	There was a positive role for probiotics in the prevention of spontaneous premature births associated with bacterial vaginosis
Ho et al. (2016) [28]	Taiwan	DBRCT	GBS-positive pregnant women	110, NR	*L*. *rhamnosus* GR-1; *L*. *reuteri* RC-14	O	1 × 10^9^	2 capsules/o.d.	Identical placebo/2 capsules/o.d.	Oral probiotics reduced the vaginal and GBS colonization rate in pregnant women
Olsen et al. (2017) [29]	Australia	Pilot RCT	GBS-positive pregnant women	34, NR	*L*. *rhamnosus* GR-1; *L*. *fermentum/reuteri* RC-14	O	10^8^	1 dose/o.d./3 wks or until the childbirth	NR	No significant difference was found in the vaginal GBS rates between the control and intervention groups. The vaginal commensals in the probiotics group were increased
Russo et al. (2018) [30]	Germany	RDBPCT	Vaginal GBS	40, NR	*L*. *acidophilus* GLA-14, LMGS-29159; *L*. *rhamnosus* HN001, AGALNM07/09514	O	5 × 10^9^	1 capsule/o.d./15 d	Maltodextrin/100 mg/o.d./15 d	Lactobacilli/lactoferrin mixtures produced significant vaginal lactobacilli colonization. Such colonization is correlated with the restoration of standard Nugent score (values 0–3) and an improvement in symptoms of AVM, including itching and discharge
Tomusiak et al. (2015) [31]	Poland	RDBPCT	Women who needed to rebalance/or restore their vaginal microbiota	160, 30.1	*L*. *fermentum* 57A; *L*. *plantarum* 57B; *L*. *gasseri* 57C	V	>10^5^	1 capsule/o.d./7 d	Identical placebo/o.d./7 d	Administration of vaginal probiotics contributed to a significant decrease in both vaginal pH and Nugent score and a significant increase in the abundance of *Lactobacillus* spp. between visits
Yang et al. (2020) [32]	Canada	RDBPCT	Pregnant women	86, 34.1 (3.7)	*L*. *rhamnosus* GR-1; *L*. *reuteri* RC-14	O	2.5 × 10^9^	1 capsule/b.i.d./12 wks	Identical placebo/b.i.d./12 wks	There was no difference in the Shannon Diversity Index between the probiotic and the placebo groups. IL-4 in the placebo group and IL-10 in both the probiotic and placebo groups were increased
Vujic et al. (2012) [33]	Croatia	RDBPCT; multicentric	BV, candidiasis, trichomoniasis, or combined	544, 32.7	*L*. *rhamnosus* GR-1; *L*. *reuteri* RC-14	O	>10^9^	1 capsule/o.d./6 wks	Identical placebo/o.d./6 wks	Oral probiotics could be an alternative, side-effect-free treatment for BV, candidiasis, and trichomoniasis combining metronidazole
Laue et al. (2017) [34]	Germany	RDBPCT; MC	BV	36, 35.8 (12.1)	*L*. *delbrueckii* subsp. *bulgaricus*; *S*. *thermophilus*; *L*. *crispatus* LbV 88; *L*. *gasseri* LbV 150 N; *L*. *jensenii* LbV 116; *L*. *rhamnosus* LbV96	O	1 × 10^7^	Yogurt drink/125 g/b.i.d./4 wks	125 g chemically acidified milk without bacterial strains/b.i.d./4 wks	Additional intake of yogurt containing probiotic strains improved the recovery rate and symptoms of BV and improved the vaginal microbiota
Ehrström et al. (2010) [35]	Sweden	RDBPCT	BV and/or VVC	95, 31.4 (7.6)	*L*. *gasseri* LN40; *L*. *fermentum* LN99; *L*. *casei* subsp. *rhamnosus* LN113; *P*. *acidilactici* LN23	V	10^8^ to 10^10^	1 capsule/b.i.d./5 d	Identical placebo b.i.d./5 d	Vaginal administration of probiotics after conventional treatment of BV and/or VVC led to the vaginal colonization of lactobacilli, fewer recurrences, and less malodorous discharge
Barthow et al. (2016) [36]	New Zealand	RDBPCT	Pregnant women	400, NR	*L*. *rhamnosus* HN001	O	6 × 10^9^	1 capsule/o.d./14–16 wks	Maltodextrin/6 months	Maternal supplementation with probiotics during pregnancy and breastfeeding could reduce rates of eczema and atopic sensitization in infants until 1 year and reduce maternal rates of gestational diabetes, BV, vaginal carriage of GBS before birth, and maternal depression and anxiety postpartum

RCT: randomized controlled trial; MC: monocentric; DB: double-blind; RDBPCT: randomized double-blind placebo-controlled trial; RDTPCT: randomized triple-blind placebo-controlled trial; DBRCT: double-blind randomized controlled trial; o.d: once daily; b.i.d.: twice daily; t.i.d.: three times daily; d: days; wks: weeks; yr: year; NR: not reported; GBS: Group B Streptococcus; O: orally; V: vaginally; AVM: abnormal vaginal microbiota.

**Table 3:** Clinical trials on pre/probiotics combined with antibiotics for the treatment of bacterial vaginosis.

Reference	Region	Study design	Participant's characteristics	No. of participants, mean age (SD)	Pre/probiotics	Dose (CFU)	Administration route	Intervention(s) combined with pre/probiotics	Control used and therapy duration	Outcomes
Bradshaw et al. (2012) [37]	Australia	RDBPCT	BV	450, 27	*L*. *acidophilus* KS400 (containing 0.03 mg oestriol)	≥10^7^	V	400 mg MTZ/b.i.d./O/7 d; CLI cream/o.d./V/7 d; vaginal pessary/12 d	Placebo/V/12 nights	Combining the oral MTZ with vaginal CLI or oral MTZ with an extended course of a vaginal probiotic did not reduce BV recurrence
Donders et al. (2010) [38]	Poland	Randomized, single-blind, active-controlled pilot study; multicentric	Premenopausal women with BV	46, 33.7 (8.9)	*L*. *acidophilus* (containing 0.03 mg oestriol)	≥10^7^	V	MTZ/500 mg/V/o.d./6 d; probiotic/o.d./6 d	NR	Lactobacilli in combination with low-dose estriol were equivalent to MTZ in the short-term treatment of BV but had less effect after 1 month
Heczko et al. (2015) [39]	Poland	RDBPCT; multicentric	BV/AV	241, NR	*L*. *gasseri* 57 C; *L*. *fermentum* 57 A; *L*. *plantarum* 57 B	≥10^8^	O	MTZ/500 mg/orally/b.i.d./7 d; probiotic/b.i.d./10 d	Identical placebo/b.i.d./10 d	Oral probiotics lengthened the remission time in patients with recurrent BV/AV and improved clinical and microbiological parameters
Hamid et al. (2013) [40]	Indonesia	Experimental	Vaginal discharge	50, NR	NR	NR	O	MTZ/b.i.d./7 d; probiotic/o.d./7 d	NR	There was no significant difference in the recovery rate of BV between the probiotic and control groups
Hakimi et al. (2017) [41]	Iran	Triple-blind RCT	BV	100, 34 (0.9)	Prebiotic vaginal gel	5 mg	V	Prebiotic vaginal gel/5 mg/o.d./7 d; MTZ tablets/250 mg/orally/t.i.d./7 d	5 mg placebo vaginal gel/o.d./7 d; MTZ tablets/250 mg/orally/t.i.d./7 d	Adjuvant treatment using prebiotic vaginal gel increased the efficacy of treatment of BV with oral MTZ
Ling et al. (2012) [42]	China	Cohort CT	BV	121, NR	*L*. *delbrueckii* subsp. *lactis* DM8909	≥10^9^	V	MTZ/500 mg/vaginally/o.d./7 d; probiotic/o.d./7 d	NR	Both MTZ and probiotics had good efficacies against BV, but probiotics maintained the normal vaginal microbiota longer due to effective and steady vaginal microbiota restoration
Marcotte et al. (2019) [43]	South Africa	Partially randomized, exploratory pilot study	BV	39, NR	*L*. *rhamnosus* DSM 14870; *L*. *gasseri* DSM 14869	1 × 10^8^	V	Cefixime/400 mg stat/doxycycline/100 mg/orally/b.i.d./7 d; MTZ/2 g stat; probiotic/o.d./30 d	NR	Probiotic did not improve BV cure rates nor alleviate recurrence, which could be due to the treatment failure or very limited power of the study
Wijgert et al. (2020) [44]	Rwanda	RCT	BV, VVC, and trichomoniasis	68, 31	*B*. *bifidum* W28; *L*. *acidophilus* W70; *L*. *helveticus* W74; *L*. *brevis* W63; *L*. *plantarum* W21; *L*. *salivarius* W24; *L. rhamnosus* (GynLP)	1.5 × 10^9^ 1.6 × 10^9^	V	MTZ/orally/2 months; probiotic/2 months	NR	It was demonstrated that the pre/probiotic treatment effectively restored the normal vaginal microbiota in 32% of the women, and 47% of the women had an improved Nugent score (*p* = 0.0001). 20 % of the subjects were unable to clear up BV after treatment (*p* > 0.05)
Palma et al. (2018) [45]	Italy	Pilot study	BV or vaginitis with concomitant HPV	117, 30.7 (9.7)	*L*. *rhamnosus* BMX	10^4^	V	MTZ/500 mg/orally/b.i.d./7 d; FCZ/150 mg/orally/o.d./2 d Short term: probiotic/o.d./10 d q72 h/20 d q5 day/2 month Long term: probiotics/q7 day/5 months	NR	Long-term probiotic users were twice as likely to solve HPV-related cytological anomalies as short-term probiotic users. Additionally, a total HPV clearance was found in 11.6% of short probiotics patients users, compared to the 31.2% of long-term vaginal lactobacilli users
Palma et al. (2018)[46]	Indonesia	True experimental	BV	24, NR	Prebiotic (GMH)	300 mg	V	MTZ/500 mg/orally/b.i.d./7 d; BV gel/o.d./vaginally/7 d; prebiotic/t.i.w./21 d	NR	The GMH and BV gel were able to reduce Nugent scores and increase Treg cell presentation and TGF-*β* levels

RCT: randomized controlled study, MC: monocentric, CT: clinical trial; RDBPCT: randomized double-blind placebo-controlled trial, o.d: once daily, b.i.d.: twice daily, t.i.d.: three times daily, d: days, wks: weeks, MTZ: metronidazole, CLI: clindamycin, FCZ: fluconazole AV: aerobic vaginitis, VVC: vulvovaginal candidiasis, GDM: gestational diabetes mellitus, NR: not reported, T.I.W: three times a week; q72h: once every 3 days, q5day: every 5 days, q7day: every 7 days, GMH: glucomannan hydrolysates, O: orally; V: vaginally, and HPV: human papilloma virus.

## Data Availability

All relevant data are included in the paper.

## Supplementary Materials

Supplementary Materials Table S1: Risk of bias assessment using a Joanna Briggs Institute (JBI) Critical Appraisal Checklist tool to assess studies in the systematic review.
