# Supplementary material for: Effects of Pro/Prebiotics Alone over Pro/Prebiotics Combined with Conventional Antibiotic Therapy to Treat Bacterial Vaginosis: A Systematic Review
Source: Int J Clin Pract. 2022 Apr 21;2022:4774783. doi: 10.1155/2022/4774783 (PMC9159122; doi:10.1155/2022/4774783)
Supplement: Supplementary Materials — Table S1: Risk of bias assessment using a Joanna Briggs Institute (JBI) Critical Appraisal Checklist tool to assess studies in the systematic review. [file 4774783.f1.docx]

**Supporting information**

Table S1: Risk of bias assessment using a Joanna Briggs Institute (JBI) Critical Appraisal Checklist tool for assessing studies in the systematic review

| Questions/  Author | 1. Was true randomization used for assignment of participants to treatment groups? | 2. Was allocation to treatment groups concealed? | 3. Were treatment groups similar at the baseline? | 4. Were participants blind to treatment assignment? | 5. Were those delivering treatment blind to treatment assignment? | 6. Were outcomes assessors blind to treatment assignment? | 7. Were treatment groups treated identically other than the intervention of interest? | 8. Was follow up complete and if Nt, were differences between groups in terms of their follow up adequately described and analyzed? | 9. Were participants analyzed in the groups to which they were randomized? | 10. Were outcomes measured in the same way for treatment groups? | 11. Were outcomes measured in a reliable way? | 12. Was appropriate statistical analysis used? | 13. Was the trial design appropriate, and any deviations from the standard RCT design (individual randomization, parallel groups) accounted for in the conduct and analysis of the trial? | Risk of Bias |
| --- | --- | --- | --- | --- | --- | --- | --- | --- | --- | --- | --- | --- | --- | --- |
| Tomusiak et al. 2015 | Y | Y | Y | Y | Y | Y | Y | Y | Y | Y | Y | Y | Y | Low |
| Bradshaw et al. 2012 | Y | Y | Y | Y | Y | Y | Y | Y | Y | Y | Y | Y | Y | Low |
| Gille et al. 2016 | Y | Y | Y | Y | Y | Y | Y | Y | Y | Y | Y | Y | Y | Low |
| Barthow et al. 2016 | Y | Y | Y | Y | Y | N | Y | Y | Y | Y | Y | Y | Y | Low |
| Donders et al. 2010 | Y | Y | Y | Y | N | N | Y | Y | Y | Y | Y | Y | Y | Low |
| Ehrstrom et al. 2010 | Y | Y | Y | Y | Y | N | Y | Y | Y | Y | Y | Y | Y | Low |
| Marcotte et al. 2019 | Y | N | Y | N | N | N | Y | Y | Y | Y | Y | Y | Y | Low |
| Hemalatha et al. 2012 | Y | Y | Y | Y | Y | Y | Y | Y | Y | Y | Y | Y | Y | Low |
| Husain et al. 2020 | Y | Y | Y | Y | Y | Y | N | Y | Y | Y | Y | Y | Y | Low |
| Indarti et al. 2018 | Y | Y | Y | Y | Y | Y | Y | Y | Y | Y | Y | Y | Y | Low |
| Laue et al. 2017 | Y | Y | Y | Y | Y | N | Y | Y | Y | Y | Y | Y | Y | Low |
| Krauss-Silva et al. 2011 | Y | Y | Y | Y | Y | Y | Y | Y | Y | Y | Y | Y | Y | Low |
| Ling et al. 2013 | Y | N | Y | N | N | N | Y | Y | Y | Y | Y | Y | Y | Low |
| Ming Ho et al. 2916 | Y | Y | Y | Y | Y | Y | Y | Y | Y | Y | Y | Y | Y | Low |
| Olsen et al. 2017 | Y | Y | Y | Y | Y | Y | Y | Y | Y | Y | Y | Y | Y | Low |
| Palma et al. 2017 | Y | N | Y | Y | N | N | Y | Y | Y | Y | Y | Y | Y | Low |
| Heczko et al. 2015 | Y | Y | Y | Y | Y | Y | Y | Y | Y | Y | Y | Y | Y | Low |
| Russo et al. 2018 | Y | Y | Y | Y | Y | Y | Y | Y | Y | Y | Y | Y | Y | Low |
| Vujic et al. 2013 | Y | Y | Y | Y | Y | Y | Y | Y | Y | Y | Y | Y | Y | Low |
| van de Wijgert et al. 2020 | Y | N | Y | N | N | N | Y | Y | Y | Y | Y | Y | Y | Low |
| Yang et al. 2020 | Y | Y | Y | Y | Y | Y | Y | Y | Y | Y | Y | Y | Y | Low |
| Hakimi et al. 2017 | Y | Y | Y | Y | Y | Y | Y | Y | Y | Y | Y | Y | Y | Low |
| Hamid et al. 2013 | Y | N | Y | N | N | Y | Y | Y | Y | Y | Y | Y | Y | Low |
| RetNningrum et al. 2018 | Y | N | Y | N | N | Y | Y | Y | Y | Y | Y | Y | Y | Low |
